# The *yfhQ *gene of *Escherichia coli *encodes a tRNA:Cm32/Um32 methyltransferase

**DOI:** 10.1186/1471-2199-7-23

**Published:** 2006-07-18

**Authors:** Elzbieta Purta, Françoise van Vliet, Karolina L Tkaczuk, Stanislaw Dunin-Horkawicz, Hirotada Mori, Louis Droogmans, Janusz M Bujnicki

**Affiliations:** 1 Laboratory of Bioinformatics and Protein Engineering, International Institute of Molecular and Cell Biology, ul. ks. Trojdena 4, 02-109 Warsaw, Poland; 2 Institute of Biochemistry and Biophysics PAS, Pawinskiego 5a, 02-106 Warsaw, Poland; 3 Institut de Recherches Microbiologiques Wiame, avenue E. Gryson 1, B-1070 Bruxelles, Belgium; 4 Institute of Technical Biochemistry, Technical University of Lodz, B. Stefanowskiego 4/10, 90-924 Lodz, Poland; 5 Institute of Advanced Biosciences, Keio University, Tsuruoka, Yamagata 997-0035, Japan; 6 Laboratoire de Microbiologie, Université Libre de Bruxelles, Institut de Recherches Microbiologiques Wiame, avenue E. Gryson 1, B-1070 Bruxelles, Belgium; 7 Institute of Molecular Biology and Biotechnology, Adam Mickiewicz University, Umultowska 89, 61-614 Poznan, Poland

## Abstract

**Background:**

Naturally occurring tRNAs contain numerous modified nucleosides. They are formed by enzymatic modification of the primary transcripts during the complex RNA maturation process. In model organisms *Escherichia coli *and *Saccharomyces cerevisiae *most enzymes involved in this process have been identified. Interestingly, it was found that tRNA methylation, one of the most common modifications, can be introduced by *S*-adenosyl-L-methionine (AdoMet)-dependent methyltransferases (MTases) that belong to two structurally and phylogenetically unrelated protein superfamilies: RFM and SPOUT.

**Results:**

As a part of a large-scale project aiming at characterization of a complete set of RNA modification enzymes of model organisms, we have studied the *Escherichia coli *proteins YibK, LasT, YfhQ, and YbeA for their ability to introduce the last unassigned methylations of ribose at positions 32 and 34 of the tRNA anticodon loop. We found that YfhQ catalyzes the AdoMet-dependent formation of Cm32 or Um32 in tRNA^Ser1 ^and tRNA^Gln2 ^and that an *E. coli *strain with a disrupted *yfhQ *gene lacks the tRNA:Cm32/Um32 methyltransferase activity. Thus, we propose to rename YfhQ as TrMet(Xm32) according to the recently proposed, uniform nomenclature for all RNA modification enzymes, or TrmJ, according to the traditional nomenclature for bacterial tRNA MTases.

**Conclusion:**

Our results reveal that methylation at position 32 is carried out by completely unrelated TrMet(Xm32) enzymes in eukaryota and prokaryota (RFM superfamily member Trm7 and SPOUT superfamily member TrmJ, respectively), mirroring the scenario observed in the case of the m^1^G37 modification (introduced by the RFM member Trm5 in eukaryota and archaea, and by the SPOUT member TrmD in bacteria).

## Background

**Figure 1:**
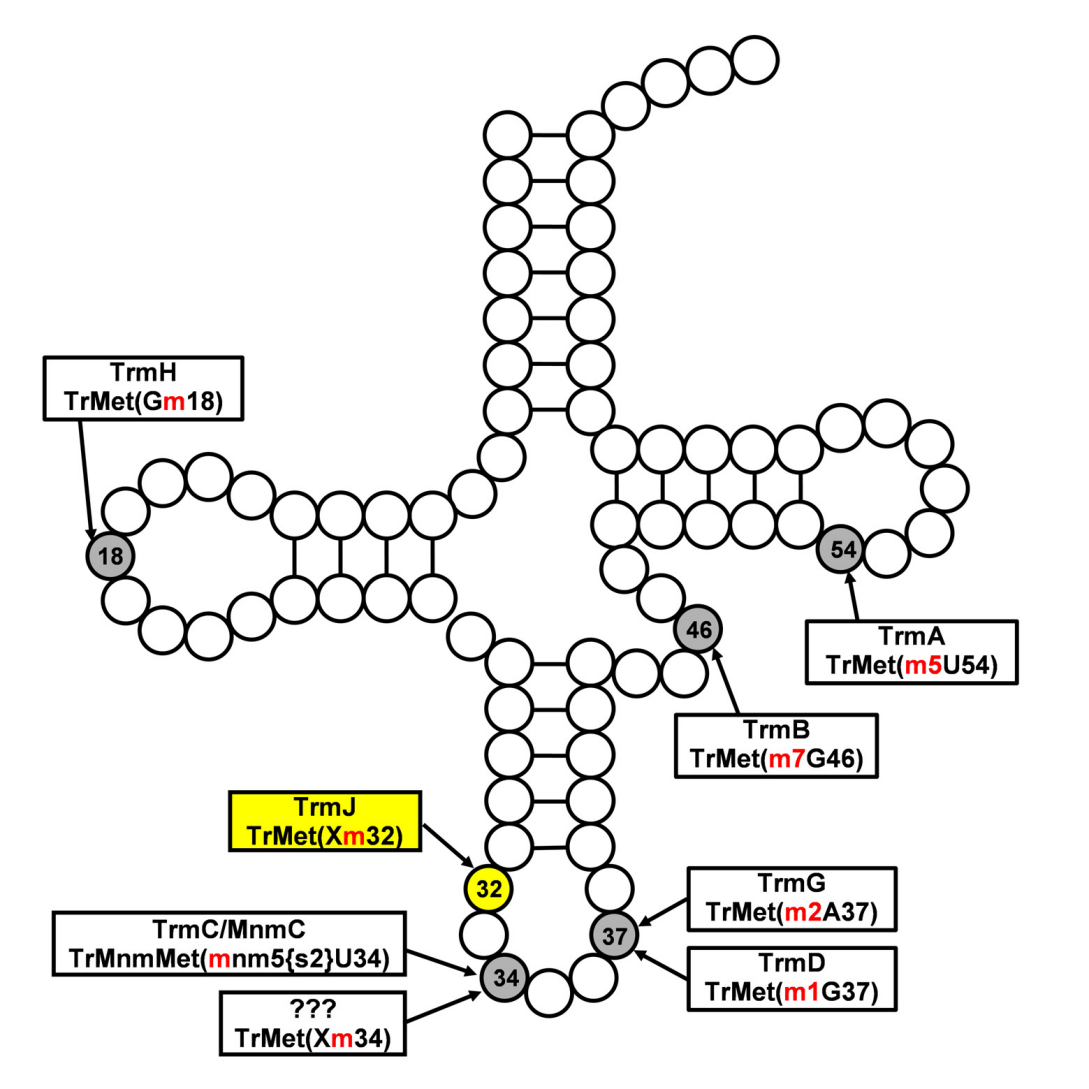
The cloverleaf representation of tRNAs from *E. coli*, with known methylated nucleosides and the respective enzymes indicated.

All mature transfer RNAs (tRNA) molecules, from all known living organisms, contain numerous modified nucleosides at multiple positions [1]. Although other RNA species also contain modified nucleosides, they are less common than in tRNA, where over 80 modifications have been found to occur at typically ~10%, but sometimes even at as many as 25% positions [2]. Recently, owing to the availability of complete genome sequences, there has been a remarkable progress in identification of enzymes that introduce modifications in tRNAs of model organisms *Escherichia coli *and *Saccharomyces cerevisiae *(reviews: [3,4]). For instance, as a part of a large-scale project, aiming at identification of the complete repertoire of RNA methyltransferases (MTases) in *E. coli *by combination of bioinformatics and experimental analyses, we have recently identified the so far "missing" enzymes that introduce m^7^G46 [5] and mnm^5^s^2^U34 [6]. In *E. coli *the only tRNA methylations, for which the corresponding enzymes (and genes that encode them) remain unknown, are those of the 2'-OH groups of ribose at positions 32 and 34 of the anticodon loop (Figure 1).

Ribose methylation is one of the most common modifications. In *E. coli *it was found to be introduced at different positions of different RNAs by site-specific enzymes, *S*-adenosyl-L-methionine (AdoMet)-dependent MTases that belong to two structurally and phylogenetically unrelated protein superfamilies: Rossmann-fold MTase (RFM) [7,8] and SPOUT [9]. These superfamilies have been defined based on sequence and structural comparisons (review: [10]). The RFM superfamily can be exemplified by the 23S rRNA:Um2552 MTase RrmJ (which is also able to methylate tRNA in vitro, albeit at an unspecified position) [11,12], while the SPOUT superfamily can be exemplified by the tRNA:Gm18 MTase TrmH [13,14].

Previously, we found that in *S. cerevisiae *methylations at positions 32 and 34 are introduced by the Trm7 enzyme, a close homolog of RrmJ and a member of the RFM superfamily [15]. However, apart from the rRNA MTase RrmJ, there are no close homologs of Trm7 in *E. coli *that could carry out the corresponding reactions in tRNAs [16], which suggests that modifications at positions 32 and/or 34 in prokaryotic and eukaryotic tRNAs could be carried out by analogous, i.e. unrelated proteins. To test this hypothesis, we assayed the so far functionally uncharacterized members of the SPOUT superfamily [9]: YibK, LasT, YfhQ, and YbeA for their ability to exert methylation of tRNA at positions 32 and/or 34.

## Results and discussion

### Preparation of the substrates and methyltransferase candidates

Analysis of the tRNA sequences and modified nucleosides in the MODOMICS database [17] revealed the presence of 2'-O-methylated uridine (Um) in position 32 of the anticodon loop in tRNA^Gln1 ^(UUG) and tRNA^Gln2 ^(CUG), and 2'-*O*-methylated cytidine (Cm) at the same position in tRNA_f_^Met1 ^(CAU), tRNA_f_^Met2 ^(CAU), tRNA^Ser1 ^(UGA), and tRNA^Trp1 ^(CCA). On the other hand, the nucleoside in position 34 was found to be 2'-*O*-methylated in tRNA^Leu4 ^(UAA) to 5-carboxymethylaminomethyl-2-*O*-methyluridine (cmnm^5^Um), and in tRNA^Leu5 ^(CAA) to Cm. We selected tRNA^Ser1 ^and tRNA^Gln2 ^as representative substrates for the methylation at position 32, while tRNA^Leu5 ^was selected as a representative substrate for the methylation at position 34. Transcripts were generated *in vitro *using T7-RNA polymerase (see Methods).

**Figure 2:**
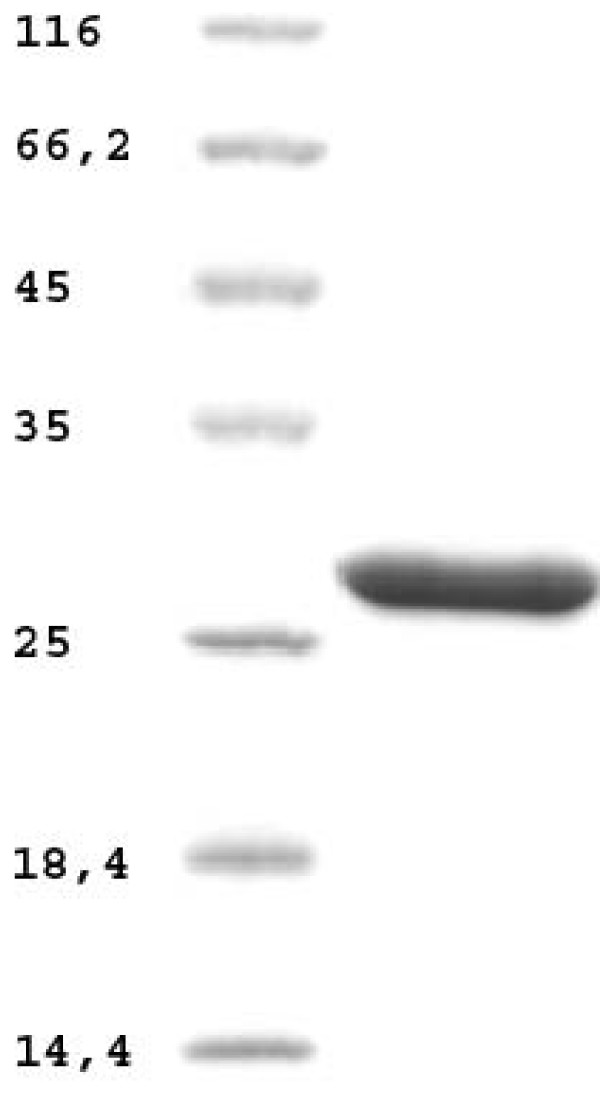
**SDS-PAGE analysis of the purified YfhQ protein**. Left lane, molecular mass markers (Pharmacia-Biotech). Right lane, purified recombinant YfhQ.

**Figure 3:**
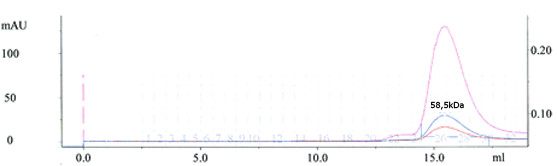
**Gel-filtration profile of the purified YfhQ protein**. Magenta line: UV 232 nm, red: UV 260 nm, blue: UV 280 nm. The peak at 16 ml corresponds to a dimeric form of YfhQ.

Among the cloned open reading frames without experimentally assigned functions, we selected a set of putative MTases identified as candidates for RNA modification enzymes in earlier studies: YibK, LasT, YfhQ, and YbeA [9,18]. All these proteins are members of the SPOUT superfamily of MTases [9], which is characterized by the presence of a deep topological knot [19,20]. The experimental analysis of these putative tRNA modification enzymes in *E. coli *was greatly facilitated by the availability of a complete set of cloned individual genes encoding His-tagged proteins [21] and the corresponding knock-out (K.O.) strains [22], which should lack the corresponding MTase activities and hence, contain unmethylated substrates. Thus, on the one hand, total tRNA was extracted from the *yibK*, *lasT*, *yfhQ*, and *ybeA *K.O. strains. On the other hand, we have expressed and purified His-tagged YibK, LasT, YfhQ, and YbeA proteins. Here, data is shown in detail only for YfhQ, as it was the only MTase candidate, for which we were able to demonstrate the tRNA MTase activity using the selected substrates. SDS-PAGE analysis of the fractions containing YfhQ in the presence of 7.5% β-mercaptoethanol showed one band corresponding to approximately 30 kDa (Figure 2), in agreement with the theoretical value calculated for the YfhQ monomer (29.168 kDa). The immunoblot analysis using anti-His tag antibodies confirmed the presence of a His-tagged protein in the 30 kDa band (data not shown). Interestingly, SDS-PAGE analysis in the lower concentration of β-mercaptoethanol (e.g. 5%) revealed an additional band of approximately 60 kDa, suggesting the existence of a very strong dimer. It was verified by Western blot analysis as containing the His6 protein (data not shown), this suggesting that it is indeed the dimeric form of the 30 kDa protein. Gel filtration chromatography revealed that the apparent molecular mass of the native form of the YfhQ protein is about 58 kDa (Figure 3). Thus, we conclude that YfhQ exists as a strong dimer, similarly to its homologs from the SPOUT superfamily with known structures (e.g. YibK [23]). The results of SDS-PAGE and gel filtration analyses for YibK, LasT, and YbeA were qualitatively similar, e.g. their molecular mass agreed with the theoretical values expected for monomers and dimers (data not shown).

### yfhQ encodes a MTase responsible for the formation of Cm/Um 32 in the anticodon loop of tRNA

**Figure 4:**
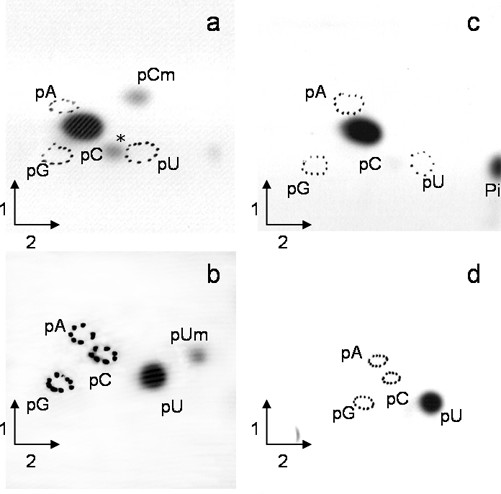
**Nuclease P1 cleavage of *in vitro *transcribed *E. coli *tRNA^Ser1 ^and tRNA^Gln2 ^incubated with YfhQ and AdoMet**. Autoradiography of two-dimensional chromatograms of 5'-phosphate and 3'-phosphate nucleosides on thin layer cellulose plates. [α-^32^P]CTP-labelled *in vitro *transcribed tRNA^Ser1 ^(2.5 × 10^5 ^cpm) (a and c) and [α-^32^P]UTP-labelled *in vitro *transcribed tRNA^Gln2 ^(2.5 × 10^5 ^cpm) (b and d) was incubated in the presence (a and b) or absence (c and d) of the YfhQ protein. The reaction mixture contained 50 mM PIPES-Na, pH 7.0, 4 mM MgCl_2_, 50 μM AdoMet and 5 μg of the purified YfhQ protein. After 90 min incubation at 37°C, the tRNA was recovered, cleaved by nuclease P1, the resulting nucleotides were analyzed as described [34]. The additional spot marked with an asterisk ("*") corresponds to an unknown species, a product either of an unusual cleavage pattern modified by the presence of 2'-O-methylation or degradation.

In order to test whether YfhQ, YibK, LasT, or YbeA could methylate the U or C nucleosides in positions 32 or C in position 34 of the anticodon loop, we incubated the purified proteins with AdoMet and with *in vitro *transcribed tRNAs: [α-^32^P]CTP-labelled tRNA^Ser1^, [α-^32^P]UTP-labelled tRNA^Gln2^, and [α-^32^P]CTP-labelled tRNA^Leu5^. Combinations of every protein with every tRNA substrate were tested. After incubation, the tRNA was hydrolyzed using nuclease P1 and the resulting 5'-phosphate nucleosides were analyzed by two-dimensional cellulose thin layer chromatography (2D-TLC) followed by autoradiography. The results shown in Figure 4 demonstrate that YfhQ is able to introduce Cm in the *in vitro *transcribed tRNA^Ser1 ^and Um in the *in vitro *transcribed tRNA^Gln2^. We were unable to detect the MTase activity of YibK, LasT, or YbeA on any of these substrates (data not shown), therefore they were not analyzed further.

**Figure 5:**
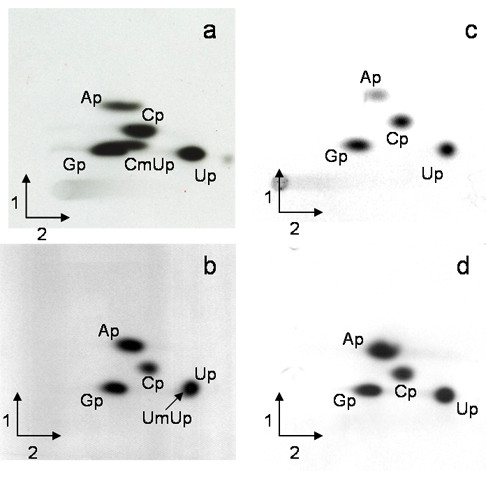
**RNAse T2 cleavage of *in vitro *transcribed *E. coli *tRNA^Ser1 ^and tRNA^Gln2 ^incubated with YfhQ and AdoMet**. Autoradiography of two-dimensional chromatograms of 5'-phosphate and 3'-phosphate nucleosides on thin layer cellulose plates. [α-^32^P]UTP-labelled *in vitro *transcribed tRNA^Ser1 ^(2.5 × 10^5 ^cpm) (a and c) and [α-^32^P]UTP-labelled *in vitro *transcribed tRNA^Gln2 ^(2.5 × 10^5 ^cpm) (b and d) was incubated in the presence (a and b) or absence (c and d) of the YfhQ protein. The reaction mixture contained 50 mM PIPES-Na, pH 7.0, 4 mM MgCl_2_, 50 μM AdoMet and 5 μg of the purified YfhQ protein. After 90 min incubation at 37°C, the tRNA was recovered, cleaved by RNAse T2, the resulting nucleotides were analyzed as described [38].

In order to further confirm that YfhQ is responsible for methylation at position 32, a similar experiment was performed using [α-^32^P]UTP-labelled tRNA^Ser1^. After incubation in the presence of AdoMet and purified YfhQ, the tRNA was hydrolyzed by ribonuclease (RNase) T2, which cleaves the phosphodiester bonds to generate 3'-monophosphate nucleosides but does not cut the phosphodiester bond between a 2'-O-methylated nucleoside and the 3'-adjacent nucleoside, leaving 2'-O-methylated dinucleotides. The analysis of cleavage products revealed the presence of radioactively labelled CmUp (Figure 5), demonstrating that YfhQ methylates a C nucleoside 5'-adjacent to a U. A similar experiment performed using [α-^32^P]UTP-labelled tRNA^Gln2 ^revealed the formation of radioactively labelled UmUp (Figure 5). These results are perfectly consistent with the expected 2'-O-methylation at position 32 (followed by U both in tRNA^Ser1 ^and tRNA^Gln2^).

**Figure 6:**
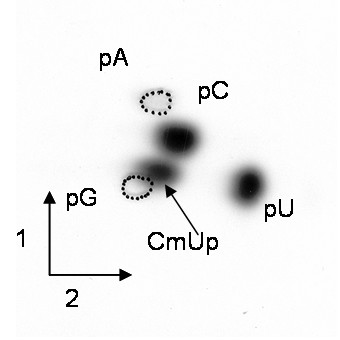
**Complete RNAse T1 cleavage followed by nuclease T2 cleavage of *in vitro *transcribed *E. coli *tRNA^Ser^^1 ^incubated with YfhQ and AdoMet**. Autoradiography of a two-dimensional chromatogram of 5'-phosphate nucleosides on a thin layer cellulose plate. [α-32P]CTP-labelled *in vitro *transcribed tRNA^Ser^^1 ^(1.5 × 10^6 ^cpm) was incubated with the YfhQ protein. The reaction mixture contained 50 mM PIPES-Na, pH 7.0, 4 mM MgCl_2_, 50 μM AdoMet and 5 μg of the purified YfhQ protein. After 90 min incubation at 37°C, the tRNA was recovered, digested overnight at 37°C by 400 u RNAse T1 in 100 mM Tris-HCl pH 7.5. The resulting oligonucleotides were separated on a 20% polyacrylamide gel (19:1) and revealed by autoradiography. Oligonucleotides were eluted from the gel and cleaved by nuclease T2. The resulting nucleotides were analyzed as described [38]. This image shows the TLC analysis for a 5-nucleotide long fragment (nucleotides 31–35).

We confirmed the localization of the 2'-O-methyl group by an independent approach. [α-32P]UTP-labelled tRNA^Ser^^1 ^was modified using purified YfhQ and then cleaved by RNAse T1. The resulting oligonucleotides (8, 6, 5 and 4-nt long fragments) were separated on a 20% polyacrylamide gel and revealed by autoradiography. The oligonucleotides were eluted from gel and cleaved with RNAse T2. The TLC analysis of cleavage products revealed the presence of radioactively labelled CmUp (Figure 6) derived from a 5-nt fragment, demonstrating that YfhQ methylates a C nucleoside 5'-adjacent to a U nucleoside in a given nucleotide context, encompassing position 32.

**Figure 7:**
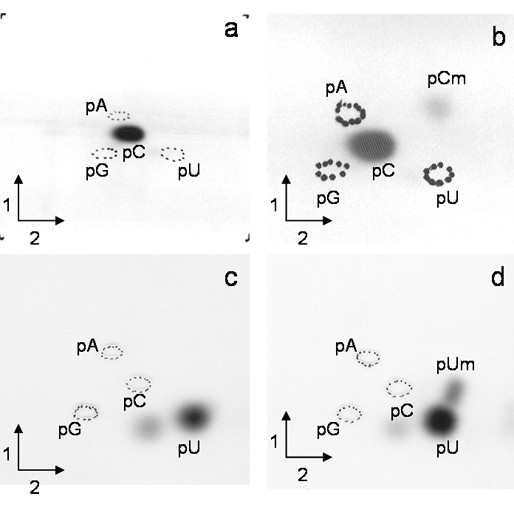
**Nuclease P1 cleavage of *in vitro *transcribed *E. coli *tRNA^Ser1 ^and tRNA^Gln2 ^incubated with cellular extracts and AdoMet**. Autoradiography of two-dimensional chromatograms of 5'-phosphate nucleosides on thin layer cellulose plates. [α-^32^P]CTP-labelled *in vitro *transcribed tRNA^Ser1 ^(2.5 × 10^5 ^cpm) (a, b) and [α-^32^P]UTP-labelled *in vitro *transcribed tRNA^Gln2 ^(2.5 × 10^5 ^cpm) (c, d) was incubated with a crude extract of the *YfhQ* K.O. strain (a and c), or of the MC1061 *E. coli *strain (b and d). The reaction mixture contained 50 mM PIPES-Na, pH7.0, 4 mM MgCl_2_, 50 μM AdoMet and 100 μg of total protein. After 90 min incubation at 37°C, the tRNA was recovered, digested by nuclease P1 and the resulting nucleotides were analyzed as described [38].

**Figure 8:**
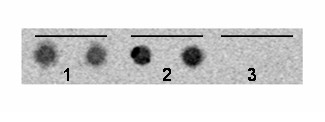
**Dot-blot analysis of total tRNA from the *yfhQ *K.O. strain incubated with YfhQ and AdoMet**. The reaction mixture containing 50 mM PIPES-Na, pH 7.0, 4 mM MgCl_2_, 100 μg of total tRNA from *yfhQ *K.O. strain, 5 μg of the purified YfhQ protein (1-YfhQ protein purified from the *yfhQ *K.O. strain transformed with a plasmid carrying the *yfhQ *gene, 2 – YfhQ purified from the regular overexpressing strain, 3 – a negative control experiment without any protein), and 10 μM [^14^C-*methyl*] AdoMet (53 mCi/mmol). After 60 min incubation at 37°C, 50 μl aliquots were filtered in duplicate in a dot-blot manifold (Scie-Plast) using a DE-81 cellulose filter. The filter was washed three times with 200 μl of the reaction buffer and one time with 70% EtOH, dried and exposed to the PhosphorImager screen. The resulting image was scanned on the Storm 820 PhosphorImager.

To determine whether the Cm32/Um32 MTase activity was affected in the *yfhQ *K.O. strain, cell extracts of *E. coli *MC1061 and of *yfhQ *K.O. strains were incubated with AdoMet and [α-^32^P]CTP-labelled *in vitro *transcribed tRNA^Ser1 ^or [α-^32^P]UTP-labelled tRNA^Gln2^. After incubation, tRNA was hydrolyzed by nuclease P1 and the nucleotides were analyzed by 2D-TLC and autoradiography. The results shown in Figure 7 revealed the absence of Cm/Um formation in *yfhQ *K.O. extract. This result was complemented by transforming the *yfhQ *K.O. strain with a plasmid carrying the *yfhQ *gene, purifying the YfhQ protein from that strain, as described in the Methods section, and testing it for the MTase activity (Figure 8). The observed activity allows us to conclude that YfhQ is essential for the Cm32/Um32 methylation.

**Figure 9:**
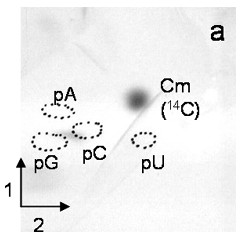
**Nuclease P1 cleavage of total tRNA from *yfhQ *K.O. strain incubated with YfhQ and AdoMet**. Autoradiography of a two-dimensional chromatogram of 5'-phosphate nucleosides on a thin layer cellulose plate. The reaction mixture containing 50 mM PIPES-Na, pH 7.0, 4 mM MgCl_2_, 100 μg of total tRNA from *yfhQ *K.O. strain, 5 μg of the purified YfhQ protein, and 10 μM [^14^C-*methyl*]AdoMet (53 mCi/mmol). After 60 min incubation at 37°C, the tRNA was recovered, digested by nuclease P1 and the resulting nucleotides were analyzed as described [38].

We also found that total (crude) tRNA extracted from the wild-type strain MC1061 was not a substrate for the purified YfhQ enzyme, while tRNA from the *yfhQ *K.O. strain was an excellent substrate for this enzyme. The purified YfhQ protein was incubated with ^14^C-radiolabelled AdoMet ([*methyl*-^14^C]AdoMet) and total tRNA extracted either from the wild type strain MC1061 (supposedly fully methylated) or from the yfhQ_K.O. strain (supposedly unmethylated in the position specific for the YfhQ MTase). After incubation, the tRNA was hydrolyzed by nuclease P1 and the resulting nucleotides were analyzed by 2D-TLC and autoradiography. As expected, the result shown in Figure 9 revealed the formation of a radioactive compound with migration characteristic for Cm only in the case of tRNA from the *yfhQ *K.O. strain, but not in the case of the fully modified tRNA. This suggests that in *E. coli *YfhQ is the only MTase responsible for the formation of Cm32 in tRNA. Curiously, no Um was detected in this last experiment. The cause could be that tRNAs that normally contain Um32 are much less abundant than tRNAs containing Cm32. However, the possibility that another MTase can also form Um32 cannot be totally excluded. In accordance with the recently proposed, uniform nomenclature for RNA modification enzymes [17] we suggest to rename YfhQ as TrMet(Xm32). Alternatively, according to the traditional nomenclature for bacterial tRNA MTases, it could be named TrmJ.

### Sequence analysis and modeling of YfhQ reveals a conserved active site common to ribose 2'-O-MTases from the SPOUT superfamily and underscores the convergent evolution with the RFM superfamily

**Figure 10:**
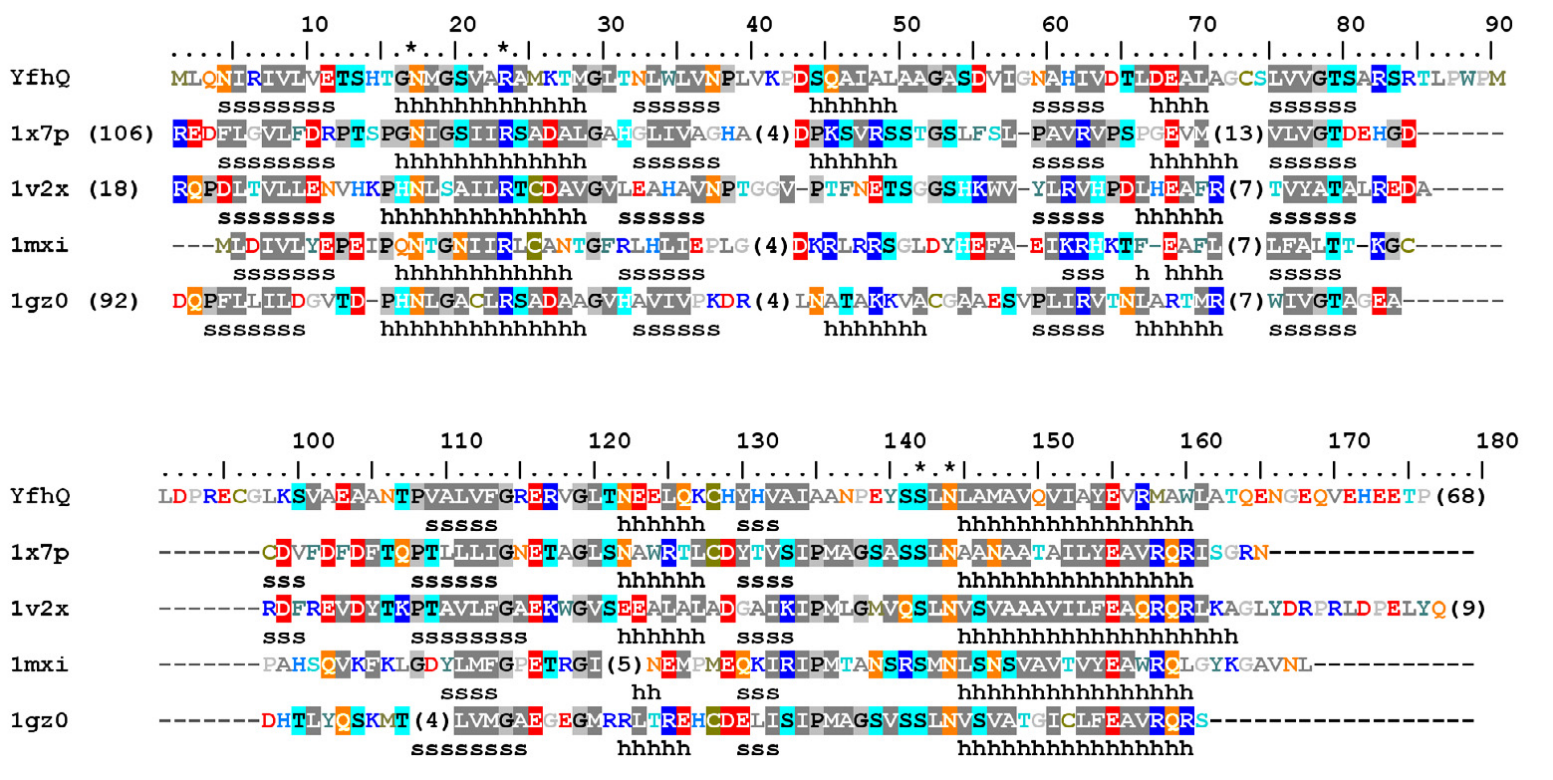
**Fold recognition alignment between YfhQ and the experimentally solved structures of SPOUT MTases**. The sequence of YfhQ has been aligned to the structures of RlmB from *Escherichia coli *(1gz0.pdb), AvirB from *Streptomyces viridochromogenes *(1x7p.pdb), TrmH from *Thermus thermophilus *(1v2x.pdb), and YibK from *Haemophilus influenzae *(1mxi.pdb), using the GeneSilico meta server [24]. Amino acid residues predicted to be involved in cofactor binding and in the methyl transfer reaction are indicated by asterisks ("*").

**Figure 11:**
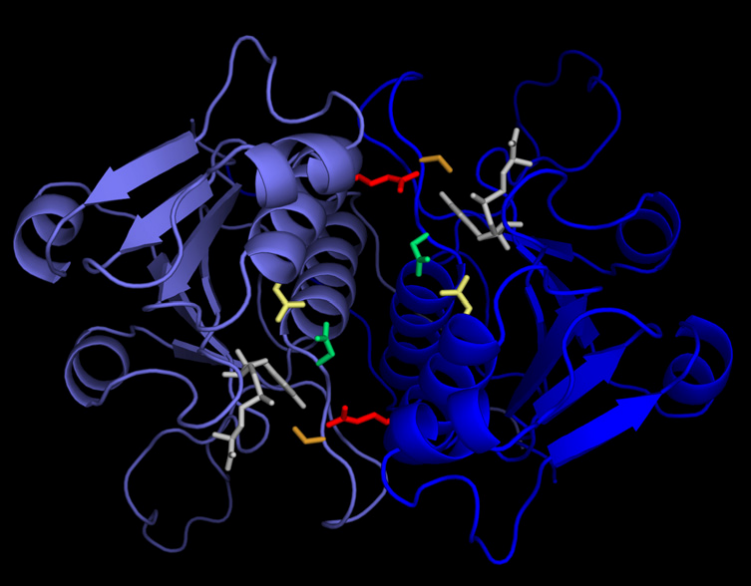
**Three-dimensional model of the YfhQ dimer in complex with AdoMet**. Both monomers are shown in the cartoon representation, in different shades of blue. AdoMet molecules (white) and residues predicted to be involved in catalysis in YfhQ and conserved in other ribose MTases (see Figure 7) are shown in the wireframe representation in different colors (N17 in yellow, R23 in red, S142 in orange, and N144 in green). The coordinates of the model are available from the corresponding author upon request.

YfhQ was previously reported to belong to the SPOUT superfamily of MTases [9], although at that time no structural information was available for any of these proteins to guide the identification of the active site. Only recently, a number of SPOUT structures were solved, providing templates for homology modeling of other members. We carried out the protein fold-recognition analysis for the YfhQ sequence using the GeneSilico meta-server [24] to predict its structure. We found, as expected, that the structures of SPOUT superfamily members were identified as the only compatible templates for YfhQ, with genuine ribose MTases RlmB [19], TrmH [14], and AviRb [25], as well as putative MTases RrmA [20] and YibK [23] reported with highest scores (Figure 10). We constructed a homology model of YfhQ using the "FRankenstein Monster's approach" [26,27]. Comparison of the model with the templates (Figure 11) reveals the common "knotted" structure of the AdoMet-binding site and conservation of the residues demonstrated to be involved in catalysis in TrmH [28]. This suggests that tRNA MTases TrmH and YfhQ use a very similar mechanism for methylation of ribose in different positions, 18 and 32, respectively. The same active site is also conserved in YibK, which we found not to methylate tRNA and which we predict to be involved in modification of one (or more) of the "unassigned" ribose methylations in rRNA [29]. We predict that the tRNA-binding activity and substrate specificity of YfhQ is at least in part dependent on the presence of the C-terminal extension, which is absent from other members of this family (e.g. no extensions in YibK) or replaced by different domains or extensions implicated in RNA-binding (e.g. additional helices at N- and C-termini present in TrmH or the N-terminal domain in RlmB and AviRb).

**Figure 12:**
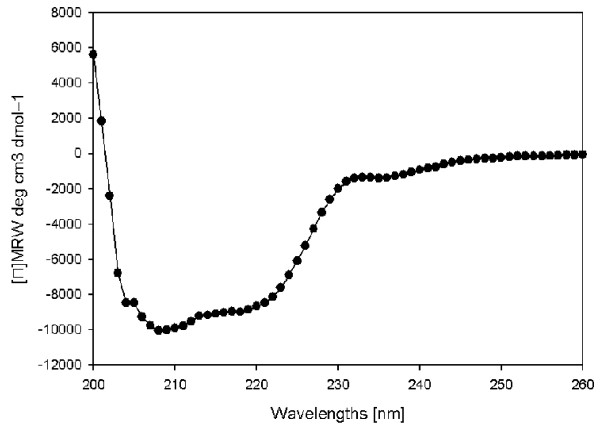
**Far UV CD spectrum of YfhQ protein**. Spectra were measured in 0.15 M Tris-HCl pH7.5 buffer and corrected by substraction of a buffer spectrum. The concentration of YfhQ protein was 6.6 μM. Three accumulations were measured, which were recorded in millidegrees every 1 nm. The mean residue ellipticities for the spectra were calculated to aid in secondary structure determination by comparison with standard spectra for secondary structure elements.

The statistical significance of sequence conservation between YfhQ and previously characterized SPOUT MTases of known structure (expectation value 3*10^-16 ^in the 2^nd ^iteration of PSI-BLAST [30]) practically guarantees that their structures are very similar. Experimental support for this prediction is obtained from the Circular Dichroim (CD) analysis (Figure 12, see Methods for details), which reveals that the secondary structure content of YfhQ (approximately 43% of helices and 23% of strands) is similar to the prediction reported here, taking into account the helical structure of the unmodeled C-terminal region. These values are also comparable to the secondary structure observed in the crystal structure of a bona fide SPOUT member YibK and our measurements of CD spectra carried out for YibK (data not shown).

## Conclusion

Our results reveal that methylation at position 32 in the anticodon loop of bacterial and eukaryotic tRNAs is carried out by completely unrelated enzymes: a SPOUT superfamily member YfhQ and RFM superfamily member Trm7. This scenario is strikingly similar to the one observed in the case of m^1^G37 modification, which is carried out by the SPOUT superfamily member TrmD in bacteria and by the RFM superfamily member Trm5 in eukaryota and archaea [31,32]. On the other hand, methylation of ribose at position 18 is carried out by members of the SPOUT superfamily both in prokaryota and eukaryota: TrmH [13], and Trm3 [33], respectively, while the *N*^1^-methylation of adenosine A58 (not observed in *E. coli*, but e.g. in *Thermus thermophilus*) is catalyzed by members of the RFM superfamily [34,35]. It is unclear which of these two MTase superfamilies was more ancient and how they replaced each other for the methylation of different positions in tRNAs from different phylogenetic lineages. Among the four members of the SPOUT superfamily studied in this work (YfhQ, YibK, LasT, and YbeA) we were unable to identify the MTase specific for the position 34 in *E. coli *tRNAs. It remains to be determined if this last missing tRNA MTase is present among the remaining, so far uncharacterized members of SPOUT and RFM superfamilies [8,9] and whether it uses a known active site or invented a new one for the same reaction. Definitely, more work is needed to elucidate the complicated pathways of evolution of RNA modification systems in all Domains of Life.

## Methods

### Preparation of the substrates

Plasmids containing the *yfhQ*, *yibK*, *lasT*, and *ybeA *genes inserted into the pCA24N vector and the corresponding *E. coli *K.O. strains were constructed as described earlier [21,22]. pBlueScript II KS (+) (Stratagene) has been modified by site-directed mutagenesis to introduce BpiI and Mph1103I cloning sites in pBlueScript II KS (+) polylinker, resulting in pKS_RNA vector. The *serT *and *leuZ *genes, encoding tRNA^Ser1 ^and tRNA^Leu5 ^respectively, were PCR-amplified from the *E. coli *genomic DNA using primers 5'-GCATGCATTGGCGGAAGCGCAGAGATTCGAAC-3' and 5'-GAAGACCCTATAGG AAGTGTGGCCGAGCGGTTG-3' for *serT *and 5'-TAATGCATGGTACCCGGAG CGGGAC-3' and 5'-AGACCCTATAGCCCGGATGGTGGAATCGGTAG-3' for *leuZ*. The final PCR product was cloned into the pKS_RNA vector, generating plasmids pKS_SerT and pKS_LeuZ. Transcripts were generated *in vitro *using T7-RNA polymerase and Mph1103I-cleaved pKS_SerT or pKS_LeuZ plasmids as templates. The tRNA^Gln2 ^transcript was generated exactly as described in [36]. Full-length transcripts were purified by 10% polyacrylamide gel electrophoresis.

### Expression and purification of the YfhQ, YibK, LasT, and YbeA recombinant proteins

Proteins were expressed in *E. coli *strain BL21 (DE3). Transformed cells were grown at 37°C in Luria broth (supplemented with chloramphenicol at 30 μg/mL) to an optical density at 660 nm (OD_660_) of 0.7. At this stage, IPTG (isopropylthiogalactopyranoside) was added up to a final concentration of 1 mM to induce recombinant protein expression. Cells were harvested after 3 hours incubation at 37°C and resuspended in buffer A (50 mM Tris-HCl, pH 7.5, 10 mM MgCl_2_, 10% glycerol) and lysed by sonication. The lysate was cleared by centrifugation (20,000 × *g *during 10 min) and was applied to a column of Chelating Sepharose Fast Flow (Pharmacia Biotech) charged with Ni^2+^. The column was washed with buffer A supplemented with 5 mM imidazole and the adsorbed material was eluted with a linear gradient (0.05 M up to 0.4 M) of imidazole. Eluted fractions were analyzed by SDS-PAGE in the presence of 5–7.5% β-mercaptoethanol. In the higher concentration of β-mercaptoethanol only the monomeric form was observed (Figure 2), while the lower concentrations allowed to observe both the monomeric and dimeric forms. YfhQ was further purified by gel filtration chromatography. The partially purified enzyme was applied on a Superdex 200 column (Pharmacia Biotech) equilibrated with buffer B (50 mM Tris-HCl, pH 7.5, 150 mM NaCl, 10 mM MgCl_2_, 10% glycerol).

### Circular Dichroism analysis

CD spectra were collected on the Jasco-810 spectropolarimeter with a temperature controller. The concentration of YfhQ protein was 5 μM and YibK protein 6.6 μM. Scans were collected at 20°C from 200 to 260, in 1 nm steps, using a 1 mm pathlength cuvette. Secondary structure content was estimated from CD spectrum using the CDpro software [37].

## Authors' contributions

EP participated in the design, execution and interpretation of all experimental analyses, preparation of the figures and writing of the manuscript. FvV participated in nucleotide analyses by thin layer chromatography. KLT carried out sequence analysis and modeling of the MTase candidates and prepared Figures 9 and 10. SDH participated in tRNA sequence analysis and prepared Figure 1. HM provided constructs expressing YfhQ, YibK, LasT, YbeA and the corresponding knock-out strains. LD coordinated the experimental analyses, and participated in the analysis and interpretation of the data. JMB conceived of the project, coordinated its execution and drafted the manuscript. All authors read and approved the final manuscript.
